# Recombinant Interferon-β in the Treatment of Polycythemia Vera and Related Neoplasms: Rationales and Perspectives

**DOI:** 10.3390/cancers14225495

**Published:** 2022-11-09

**Authors:** Hans Hasselbalch, Vibe Skov, Lasse Kjær, Morten Kranker Larsen, Trine A. Knudsen, Marko Lucijanić, Rajko Kusec

**Affiliations:** 1 Department of Hematology, Zealand University, 4000 Roskilde, Denmark; 2 Department of Hematology, University Hospital Dubrava, 10000 Zagreb, Croatia; 3 School of Medicine, University of Zagreb, 10000 Zagreb, Croatia

**Keywords:** myeloproliferative neoplasms, essential thrombocythemia, polycythemia vera, myelofibrosis, MPN, MPNs, recombinant interferon-α2 (rIFN-α2), recombinant interferon-β (rIFN-β)

## Abstract

**Simple Summary:**

The myeloproliferative neoplasms (MPNs) are chronic blood cancers characterized by elevated blood cell counts and, after decades, the development of bone marrow failure. Blood clots are common and contribute massively to the symptom burden. Treatment with interferon (IFN) alpha-2 normalizes elevated blood cell counts within weeks to months. This treatment has been used off-label over the last 30 years. Today, a novel interferon alpha-2b formulation (Besremi) is marketed for treatment of the MPN disease polycythemia vera. Another IFN formulation is interferon beta (IFN-β), which has been used for decades in the treatment of multiple sclerosis. Several studies have shown IFN-β to possess stronger anticancer capabilities than IFN alpha-2. However, only a few cancer trials have been conducted, none in patients with MPNs. In this paper, the rationales and perspectives for using IFN-β in patients with MPNs are described, and future research directions are outlined for investigating the safety and efficacy of IFN-β in MPNs.

**Abstract:**

About 30 years ago, the first clinical trials of the safety and efficacy of recombinant interferon-α2 (rIFN-α2) were performed. Since then, several single-arm studies have shown rIFN-α2 to be a highly potent anticancer agent against several cancer types. Unfortunately, however, a high toxicity profile in early studies with rIFN-α2 -among other reasons likely due to the high dosages being used-disqualified rIFN-α2, which was accordingly replaced with competitive drugs that might at first glance look more attractive to clinicians. Later, pegylated IFN-α2a (Pegasys) and pegylated IFN-α2b (PegIntron) were introduced, which have since been reported to be better tolerated due to reduced toxicity. Today, treatment with rIFN-α2 is virtually outdated in non-hematological cancers, where other immunotherapies—e.g., immune-checkpoint inhibitors—are routinely used in several cancer types and are being intensively investigated in others, either as monotherapy or in combination with immunomodulatory agents, although only rarely in combination with rIFN-α2. Within the hematological malignancies, rIFN-α2 has been used off-label for decades in patients with Philadelphia-negative chronic myeloproliferative neoplasms (MPNs)—i.e., essential thrombocythemia, polycythemia vera, and myelofibrosis—and in recent years rIFN-α2 has been revived with the marketing of ropeginterferon-α2b (Besremi) for the treatment of polycythemia vera patients. Additionally, rIFN-α2 has been revived for the treatment of chronic myelogenous leukemia in combination with tyrosine kinase inhibitors. Another rIFN formulation-recombinant interferon-β (rIFN-β)—has been used for decades in the treatment of multiple sclerosis but has never been studied as a potential agent to be used in patients with MPNs, although several studies and reviews have repeatedly described rIFN-β as an effective anticancer agent as well. In this paper, we describe the rationales and perspectives for launching studies on the safety and efficacy of rIFN-β in patients with MPNs.

## 1. Introduction

The Philadelphia-negative myeloproliferative neoplasms (MPNs) comprise essential thrombocythemia (ET), polycythemia vera (PV), and primary myelofibrosis (PMF). The MPNs are acquired stem cell disorders that develop from the early cancer stages (ET and PV) to the advanced myelofibrosis stage [1]. Before the MPN diagnosis, patients have often experienced repeated thromboembolic events for several years (e.g., 5, 10, 15, or 20 years) with concurrent elevated blood cell counts, indicating the MPNs to have existed undiagnosed for decades before eventually being diagnosed [2,3]. The MPNs are associated with the so-called driver mutations *JAK2V617F*, *CALR,* and *MPL*. The most frequent mutation is the *JAK2V617F* mutation, which is present in nearly all patients with PV and half of those with ET and PMF [4,5,6,7,8]. The *CALR* mutations are recorded in approximately 20 and 30% of ET and PMF patients, respectively [9,10,11]. Frequently, additional mutations (e.g., *DNMT3A*, *ASXL1*, *TET2*) are recorded—most often in the advanced disease stage with severe myelofibrosis [11,12].

In recent years, chronic inflammation has been proposed to be of utmost importance in the pathogenesis of MPNs [13,14,15,16,17,18,19,20,21,22,23,24,25,26,27,28,29,30], as both a trigger and a driver of clonal evolution and disease progression. In this context, MPNs have been described as “A Human Inflammation Model” and “A Human Inflammation Model for Cancer Development”, in which the malignant clone steadily expands in a vicious self-perpetuating cycle fueled by the malignant clone itself [14,15]. Accordingly, early initiation of treatment that directly targets the malignant clone-recombinant interferon-α2 (rIFN-α2) -and the concurrent chronic inflammatory state has been argued to be a prerequisite for a successful outcome of therapeutic intervention [22,31,32,33,34,35,36,37,38,39,40,41,42,43,44,45,46,47,48,49,50,51,52,53,54]. It should be noted that this “Early Interferon Intervention Concept” should preferably be started as early as possible after the MPN diagnosis to prohibit clonal evolution due to inflammation-mediated genomic instability with subclone formation and additional mutations that might confer resistance to treatment, ultimately also mediating myelofibrotic and leukemic transformation. Most recently, this “Early Interferon Concept” has been fueled by data-driven analysis of the *JAK2V617F* kinetics during treatment with IFN-α2 [50].

Hydroxyurea (HU) is the cytoreductive agent that is most frequently used in MPNs. As a DNA-synthesis inhibitor [55,56], its use has raised concern with regard to its leukemogenic potential [57,58,59,60], since long-term exposure to HU (i.e., >10 years) may be associated with an increased risk of acute myelogenous leukemia (AML) or myelodysplastic syndrome (MDS)—the latter with an inherently high risk of leukemic transformation [57,58,59,60]. Hydroxyurea does not selectively target the malignant stem cells. Likewise, another cytoreductive agent-anagrelide—does not correct the aberrant cellular machinery in MPNs but selectively reduces the elevated platelet count by interfering with the production of platelets from rapidly proliferating clonal megakaryocytes [61]. After discontinuation of HU or anagrelide, blood cell counts rapidly increase to pretreatment values within days, emphasizing that these agents have no impact on the basic molecular aberrations that elicit clonal expansion. Despite cytoreductive treatment and aspirin, a major clinical challenge in the treatment of MPNs is the substantial risk of thrombosis [1,53,62,63,64,65,66,67], and for both venous and arterial thrombosis this risk is most pronounced within the first 3 months after the MPN diagnosis [66].

During the last 30 years, recombinant interferon-α2 has been used in the treatment of MPNs, and its safety and efficacy have been convincingly demonstrated in several studies [31,32,33,34,35,36,37,38,39,40,41,42,43,44,45,46,47,48,49,50,51,52,53,54,68,69,70,71,72,73,74,75,76,77,78,79,80,81,82,83,84,85,86,87,88,89,90,91,92,93,94,95,96,97,98,99,100,101,102]. Indeed, in a subset of patients, normalization of the bone marrow and low-burden *JAK2V617F* may be obtained after prolonged treatment (about 5 years). Importantly, these effects may be sustained even 2–3 years after the discontinuation of rIFN-α2 [31,32,38,42,44]. These highly encouraging results have paved the way towards a new era where “Minimal Residual Disease” (MRD) is actually a novel treatment goal [31,32,42,44,46]. Despite being used for decades, rIFN-α2 has only recently been labeled on the market as ropeginterferon-α2b (Besremi) and indicated for PV in adults [84,98,100]. Neither PegIntron nor Pegasys have these indications, but both have been used for decades for the treatment of MPNs. PegIntron is no longer available, but Pegasys is still available as an off-label drug for the treatment of MPNs -no longer delivered by Hoffmann-La Roche Ltd. (Basel, Switzerland), but by Pharma& Schweiz GmbH (Cham, Switzerland) (Pegasys@pharmaand.com accessed on 18 February 2021), Pharma & Schweiz GmbH signed an agreement with F. Hoffmann-La Roche Ltd. to acquire the worldwide rights to Pegasys, excluding China and Japan. Thereby, patients with MPNs are secured the long-term possibility to be treated with Pegasys. Unfortunately, some patients do not tolerate Pegasys very well. Although most studies using Pegasys in MPNs have reported excellent tolerance, with only about 10–20% of patients discontinuing Pegasys due to toxicity (mainly consisting of sustained flu-like symptoms) [74,75,77,79,82,92,93,95] (for reviews see [76,78,81]), a more recent Danish study (the DALIAH trial) recorded a discontinuation rate of up to 50% after long-term use (approximately 3-year follow-up) [102].

Few studies have compared pegylated IFN-α2 with HU [98,99,100,101,102,103], and only the Danish DALIAH trial has compared these drugs in newly diagnosed MPN patients [102]. These studies have shown pegylated IFN-α2 not to be superior to HU in terms of normalizing elevated blood cell counts after 12 and 24 months. However, after 36 months, both Pegasys/PegIntron (DALIAH trial) [102] and ropeginterferon alfa-2b (Besremi) displayed convincing superiority with regard to achieving major molecular remissions, as assessed by sustained normalization of elevated blood cell counts in concert with a decline in the *JAK2V617F* allele burden [99,102]. However, as alluded to above, a dropout rate of up to 40–50% was recorded in the DALIAH trial during long-term treatment with Pegasys or PegIntron [102].

For all of the above reasons, there is an urgent need to consider whether stem-cell-targeted therapy with pegylated IFN-α2 may be accomplished by other IFN formulations than rIFN-α2. In this regard, pegylated IFN-β may be a highly relevant treatment option, since rIFN-β has been used for decades in the treatment of multiple sclerosis (MS), with an excellent safety and efficacy profile [104,105,106,107]. Furthermore, several experimental and clinical studies have provided evidence that rIFN-β is an effective antiviral agent [108,109,110,111,112], which is currently being used worldwide in the treatment of COVID-19, either as monotherapy or in combination with other antiviral agents or anti-inflammatory agents [113,114,115]. Intriguingly, rIFN-β has also demonstrated potent anticancer capabilities very similar to or even stronger than those of rIFN-α [116,117,118,119,120,121,122,123,124,125,126]. However, clinical studies of rIFN-β have been immensely overshadowed by rIFN-α2. Thus, since 1996, the therapeutic potential of rIFN-α in the treatment of cancers has been investigated in 248 trials, whereas the role of rIFN-β as an anticancer agent has only been investigated in 7 trials [126]. The potential of rIFN-β in the treatment of neuroinflammatory diseases other than MS, such as Alzheimer’s disease (AD), has also been investigated [127,128]. Since AD and MPNs share several pathogenetic mechanisms, MPNs have most recently been described as “A Human Neuroinflammation Model for The Development of Alzheimer’s Disease” [129]. Herein, after briefly depicting the successful history of rIFN-α in the treatment MPNs, we tell the story of rIFN-β in other diseases and discuss the rationales and perspectives for launching studies on the safety and efficacy of pegylated IFN-β in the treatment of MPNs.

## 2. History of Interferon-α in MPNs

In 1957, Isaacs and Lindenmann discovered a cytokine that was able to interfere with viral replication. They named this cytokine interferon (IFN) [130]. Since then, several IFN discoveries have been made, including the identification of the IFN receptor and the JAK/STAT signal transduction pathway [131,132,133,134,135]. Soon it was realized that IFNs belong to a large family, of which the type I IFN family of cytokines comprises IFN-α, IFN-β, and the less well-characterized IFNs κ, δ, ε, ζ, τ, and ω, whereas the only type II IFN is IFN-γ. IFN-α has 13 subtypes (IFN-α1, 2, 4, 5, 6, 7, 8, 10, 13, 14, 16, 17, and 21). Of all these IFNs, IFN-α2 is the one that has been studied most extensively during the last 30–40 years. It soon became apparent that IFNs had antiproliferative and anticancer activities. With the production and purification of human leucocyte IFNs by Cantell et al. [136], the avenue opened for the first clinical study in the late 1970s on the efficacy of human leucocyte IFN in four patients with multiple myeloma (MM), who were treated for 3–19 months. Remission was complete in two patients and partial in the other two [137]. A few years later, the efficacy of human leucocyte IFN was convincingly demonstrated in patients with chronic myelogenous leukemia (CML) [138], as well as in patients with ET and PV [139,140,141]. Soon after, IFN-α2 was cloned, enabling the production of large amounts of IFNs for experimental research and clinical trials. Thereafter, an exciting era of several years began, during which the safety and efficacy of rIFN-α2 was tested in a variety of cancers, including both non-hematological (with particularly successful stories in melanoma and renal-cell carcinoma) and hematological malignancies. Amongst the latter were MM, hairy-cell leukemia (HCL), CML, MPNs, the hypereosinophilic syndromes, and systemic mastocytosis (SM) (for reviews, see [34,35]). The outstanding breakthroughs achieved in the treatment of HCL and CML were historical milestones, since before the IFN era patients with HCL and CML had a dismal prognosis due to severe bone marrow failure with serious, often atypical infections (HCL) or fatal leukemic transformation within a few years from diagnosis (CML) if the patient was not a candidate for bone marrow transplantation. Thus, during treatment with rIFN-α2, long-lasting complete remissions with normalization of peripheral blood cell counts and the bone marrow were achieved in a significant proportion of patients with HCL. Furthermore, these beneficial effects were associated with a marked improvement in immune defense against infections. Likewise, in several patients with CML, rIFN-α2 induced complete and sustained cytogenetic remissions with vanishing of the Philadelphia chromosome. In a subset of patients, major molecular remissions with a sustained reduction of the *BCR-ABL1* transcript were also obtained. Therefore, over the following decades, rIFN-α2 remained the best medical treatment for CML until the targeted treatment with the tyrosine kinase inhibitor (TKI) imatinib mesylate replaced rIFN-α2 about 25 years ago, followed by other second- and third-generation TKIs (e.g., dasatinib, nilotinib, bosutinib). Very early in the IFN era, unique mechanisms of action of IFN-α2 were revealed (see below). Thus, it was demonstrated that in CML, rIFN-α2 was able to restore the adhesion of primitive progenitor cells to marrow stroma, downregulate the *BCR-ABL1* fusion gene, and activate transcriptional factors involved in the regulation of cell proliferation, maturation, and apoptosis. In addition, immune studies revealed rIFN-α2 to have very potent immune-enhancing capacity that induced the elimination of CML cells by the immune system [142,143]. A novel mechanism of action of rIFN-α2 on hematopoietic stem cells (HSCs) was described in 2009 by Essers et al., who showed that rIFN-α2 induced cell cycling in quiescent HSCs and early progenitors [144]. Soon after, they also showed HSCs to be depleted by chronic administration of rIFN-α2, implying dormant cancer stem cells to be susceptible to manipulation via an rIFN-α2-induced wakeup call, with subsequent proliferation and unmasking of the malignant stem cells and progenitors for the immune system [145]. These studies provided the impetus for similar studies on MPNs [68,69], but also for combination therapy with imatinib and rIFN-α2, as well as later studies on rIFN-α2 with other TKIs in CML [146,147,148,149]. These studies showed combination therapy with TKI and rIFN-α2 to be much more effective than single-agent therapy due to their different modes of action and biological effects.

Despite the very prominent anticancer effects of rIFN-α2 and initial studies demonstrating the safety and efficacy of rIFN-α2 in a large number of patients with MPNs (reviewed in [34,35,37,40,41,43,46,49,76,78,81]), rIFN-α2 unfortunately disappeared in the dark. However, the interest in using rIFN-α2 in MPNs has been revived in recent years due to the mounting evidence from several studies within the last 5–10 years, which have demonstrated sustained complete hematological and major molecular remissions after long-term treatment with rIFN-α2, even up to 3 years after discontinuation of IFN-α2 [31,32,38,42,44,46]. These highly encouraging results envisage MRD as a new treatment goal in MPNs, implying normalization of peripheral blood cell values and normal bone marrow architecture after long-term treatment with rIFN-α2 [45,46]. Importantly, induction of MRD by rIFN-α2 may also open a new horizon towards a cure through vaccination strategies [150,151].

## 3. The History of IFN-β and Its Neglected Role in Cancer Treatment: Lessons from the IFN-α2 Era

As alluded to above, IFN-β belongs to the type I IFN family, which only encodes a single IFN-β in contrast with 13 IFN-α subtypes. Both IFN-α and IFN-β signal through the heterodimeric IFN-α/β receptor (IFNAR), comprising the subunits IFNRA1 and IFNRA2. Whereas several studies have shown rIFN-β to be an effective antiviral agent [108,109,110,111,112,113,114,115], its role as an anticancer agent has been overshadowed by rIFN-α2, despite the fact that rIFN-β indeed exhibits similar or even perhaps better anticancer capabilities than those of rIFN-α2 [116,117,118,119,120,121,122,123,124,125,126].

The antitumor effects of both IFN-α and IFN-β were discovered by Gresser as early as 1969 [152]. Nevertheless, over the next 50 years fewer than 10 clinical trials investigated the role of IFN-β in cancer treatment [126]. The reasons for this are several but may be explained by the same factors that are undermining the use of rIFN-α2 in the treatment of cancer today, including side effects with high dropout rates even when using low doses of rIFN-α2. However, instead of exploring the mechanisms explaining the high dropout rates, which might reveal novel insights into how to administer rIFN-α2, most researchers—i.e., oncologists and hematologists—have abandoned rIFN-α2 to pursue other treatment approaches, e.g., treatment with immune-checkpoint inhibitors.

Taking into account the fact that defective tumor immune surveillance is a highly important mechanism in the development and progression of any cancer, one might wonder why rIFN-α2 in 2022 is largely being used routinely only in the treatment of patients with MPNs, whereas its use in other cancers (e.g., malignant melanoma, renal-cell carcinoma, HCL, malignant lymphoma, MM, CML) has completely abated. rIFN-α and rIFN-β are the oldest known immunomodulatory and immune-enhancing agents, with very long track records of safety and efficacy in a large number of studies in patients with a range of diseases, including viral diseases (rIFN-α2, rIFN-β), neuroinflammatory diseases (rIFN-β in MS and AD), and cancer (rIFN-α, rIFN-β). In the context of non-hematological cancers, the efficacy of rIFN-α2 was clearly demonstrated in a subset of patients with malignant melanoma and renal-cell carcinoma, but the enthusiasm for its use was dampened due to side effects, which were attributable to the high dosages used. Thus, no studies of low-dose rIFN-α2 have been performed in patients with non-hematological cancers and, accordingly, there have also been no studies with rIFN-β. Table 1 summarizes the similarities and differences between rIFN-α2 and rIFN-β with regard to clinical, biochemical, and immunological markers in MPNs and associated key questions for future studies.

## 4. Mechanisms of Action of rIFN-α and rIFN-β

rIFN-α and rIFN-β have their immunomodulatory capabilities in common, which together contribute to enhancing tumor immune surveillance and tumor killing, including the activation of several immune cells (e.g., dendritic cells, B cells, T cells, NK cells) and enhancing the expression of major histocompatibility complex (MHC) I molecules in tumor cells [118,119,120,121,122,123,124,125,126,153]. Although other mechanisms of action of rIFN-α and rIFN-β are similar in several respects, they also differ from one another. Thus, the binding affinity of IFN-β to the interferon receptor (IFNAR) is much stronger than that of IFN-α (50-fold for IFNAR1 and 1000-fold for IFNAR2) [133,154,155,156]. Furthermore, IFNAR1–IFNAR2 complex formation can be obtained by IFN-β, but not by IFN-α stimulation [154]. In the context of their anti-inflammatory effects (as alluded to above), rIFN-β has a long track record in the treatment of MS, while rIFN-α is used in the treatment of Mediterranean fever and Behcet’s disease [157,158,159], these effects may be explained by type I IFN-mediated IL-10 induction and the suppression of inflammasome-dependent IL-1 production [160]. Table 2 summarizes rationales for use of rIFN-β in MPNs, with a focus on its anticancer capabilities.

## 5. Some Key Questions on IFN-β

### 5.1. Does IFN-β Have the potential to Restore Defective Tumor Immune Surveillance in MPNs by Increasing the Frequency and Functionality of Immune Cells?

As noted above, type I IFNs exhibit strong immune-cell-enhancing capabilities, including regulation of the number and functionality of almost all immune cells (e.g., macrophages, DCs, B cells, T cells, NK cells), thereby providing a well-balanced immune response to combat cancer [118,119,120,121,122,123,124,125,126,153] (Table 2). Briefly, by upregulating the expression of tumor antigens, the tumor cells become more accessible targets for immune attack and, accordingly, tumor killing [161,162,163]. Type I IFNs activate DCs to present cancer antigens to T cells [164], which is highly important in adaptive antitumor responses [165]. Additionally, type I IFNs promote and enhance effector CD8+ T-cell cytotoxicity [166,167] and decrease regulatory T-cell function [168,169]. Importantly, type I IFNs also decrease the number of circulating myeloid-derived suppressor cells (MDSCs), which are typically elevated in patients with cancer [170,171,172,173], and also reduce their suppression of the activity of cytotoxic T cells [172,173]. Although the abovementioned immunomodulatory effects of type I IFNs have been repeatedly reported to be very similar, the anticancer effects of IFN-β have also been reported to be stronger than those of IFN-α, although comparative clinical studies have never been conducted. Taking into account that IFN-α2 is widely used today in the treatment of MPNs, we have the platform to set up such studies, including comparisons between IFN-α2 and IFN-β with regard to their safety, efficacy, and toxicity profiles as well as comparative immune cell studies (i.e., the dynamics of frequencies and functionality during treatment with IFN-α and IFN-β), gene expression studies, and studies on neutralizing IFN-α and IFN-β antibodies to obtain novel insights into the similarities and differences between the two IFN formulations and, accordingly, to open novel paths to follow for better and safer administration of these IFN formulations (see below).

### 5.2. Does IFN-β Have the Potential to Impact the Chronic Inflammatory State in MPNs?

IFN-β exhibits strong anti-inflammatory effects via several mechanisms. Thus, IFN-β has been shown to alter the production of cytokines that are involved in T-cell polarization or in inflammation, including interleukin (IL)-1β [174,175,176,177,178]. These early results have been substantiated in subsequent studies, which have demonstrated the following highly important findings [160]: (1) IFN-β suppresses the activation of caspase-1 and the intracellular pool of pro-IL-1β, thereby blocking the secretion of IL-1β. (2) IFN-β reduces the secretion of other caspase-1-dependent cytokines, such as IL-1a and IL-18, by bone-marrow-derived dendritic cells. Furthermore, IFN-β inhibits NLRP1- and NLRP3-triggered inflammasome activity and induces IL-10 production, thereby controlling IL-1b and IL-1a precursor levels [160]. Accordingly, there are reasons to believe that the treatment of MPN patients with IFN-β may not only have the potential to normoregulate elevated blood cell counts, but also dampen the chronic inflammatory state that accompanies MPNs and likely contributes to clonal expansion and evolution.

### 5.3. How Does the Chronic Inflammatory State in MPNs Impact the Efficacy of IFN-β?

Inflammatory signaling impairs cell responses to IFNs [179]. Thus, refractoriness to rIFN-α in melanoma patients has been shown to be associated with inflammation-mediated downregulation of IFN-α2AR1 [180]. Likewise, inflammation-mediated impairment of IFN-α signaling is associated with unresponsiveness to rIFN-α2a in hepatitis patients [181]. Importantly, the inflammatory cytokines tumor necrosis factor α (TNF-α) and interleukin-1α (IL-1α) stimulate IFNAR1 degradation and, accordingly, attenuate IFN-α signaling [179]. The MPNs are associated with increased circulating plasma levels of several inflammatory cytokines, including IL-1α and TNF-α [182]. Therefore, it is reasonable to assume that the chronic inflammatory state in MPNs may impair the efficacy of IFN-β. However, the much stronger binding of IFN-β to IFNAR1 (50-fold) and IFNAR2 (1000-fold) than that of IFN-α, along with the potent anti-inflammatory capacity of IFN-β, might theoretically diminish the impact of the inflammatory cytokines on IFN2AR1 degradation.

### 5.4. Rationales for Combination Treatment with rIFN β in MPNs? Lessons from the Combination of JAK1-2 Inhibitor (Jakavi) and Pegasys in MPNs, as well as Combinations of Tamoxifen and rIFN-β and of Tamoxifen, Retinoic Acid, and rIFN-β in Breast Cancer

**Combination Therapy of rIFN-β and a JAK1-2 Inhibitor?** Taking into account the fact that inflammation impairs IFN signaling due to inflammation-mediated degradation of the IFNAR, with ensuing refractoriness and intolerance to rIFN-α that can be elicited by IFN-induced, inflammation-mediated, flu-like symptoms, a combination with the potent anti-inflammatory JAK1-2 inhibitor Jakavi might alleviate refractoriness and intolerance to rIFN-α. A preliminary case report on the successful use of this drug combination in a female PV patient [39] has been confirmed in larger series of PV and MF patients who were refractory or intolerant to Pegasys monotherapy [45,47]. Although confirmatory studies are required [49], the rationales for such a combination therapy are strong [45]. Indeed, this combination therapy may be one of the most promising ever for the treatment of MPNs [22]. Since rIFN-β may have stronger anticancer efficacy than rIFN-α, including a superior anti-inflammatory potential, it is tempting to consider whether a combination of rIFN β and Jakavi may be even more efficacious.

**Combination of Tamoxifen and rIFN-β in MPN?** Tamoxifen, a selective estrogen receptor (ER) modulator, has been used for decades in the treatment and prevention of estrogen-positive breast cancer [183]. Recent experimental studies in mice have shown that hematopoietic stem cells and multipotent progenitor cells (MPPs) express ER-α. Tamoxifen-induced apoptosis has been observed in short-term HSCs and multipotent progenitors. In addition, tamoxifen altered the expression of self-renewal genes [184]. Accordingly, altogether this study convincingly showed that tamoxifen can directly regulate the proliferation and survival of hematopoietic stem cells through ER-α expressed by HSPCs [184]. Intriguingly, tamoxifen treatment blocked the development of *JAK2V617F*-induced myeloproliferative neoplasms in mice and induced apoptosis of human *JAK2V617F*+ HSPCs in a xenograft model [184]. Tamoxifen prevented the expansion of *JAK2V617F*+ HSPCs by restoring normal apoptosis levels [184]. Based on the above data, a multicenter trial of tamoxifen was launched in the UK. The preliminary results are encouraging, showing that tamoxifen is able to induce complete or partial responses with a substantial decline in the *JAK2V617F* allelic burden in a subset of patients [185]. In the context of combination therapy of tamoxifen and rIFN-β in MPNs, it is important to note that the potential of rIFN-α or rIFN-β to increase estrogen receptor expression in human breast cancer cells [186,187,188,189] and the possibility of improving tamoxifen’s efficacy through the addition of rIFN-α or rIFN-β have been addressed in several experimental and clinical studies of breast cancer over the last 25–30 years [186,187,188,189,190,191,192]. Indeed, early studies in breast cancer cell lines showed IFN-β to be highly superior to IFN-α; accordingly, IFN-β was suggested for the treatment of all breast cancers, irrespective of their steroid receptor status [187]. Interestingly, at the same time, IFN-β was also reported to exhibit greater cell growth inhibition than that produced by tamoxifen alone. This additive effect was also prevalent regardless of the receptor status of the cells [187,193]. Furthermore, during treatment with the combination of IFN- β and tamoxifen, the expression of several IFN-β-inducible genes was found to be enhanced in human breast carcinoma cell lines relative to levels induced by IFN-β alone [194]. Accordingly, the increased antitumor activity of rIFN-β when combined with tamoxifen might also be attributed to tamoxifen-mediated enhancement of the expression of interferon-stimulated genes [194]. Since tamoxifen augmented the antiproliferative activity of IFN-β in vitro as well as in vivo [195], it was concluded that this combination might act directly on tumor cells rather than indirectly on the immune system [194]. Importantly, the inhibition of tumor growth occurred independently of a functional ER- or estrogen-dependent tumor growth [194]. Based on the above lessons from translational research on the synergistic effects of combination therapy of tamoxifen and rIFN-β in breast cancer, it is intriguing to consider whether such a combination therapy might enhance the anticancer efficacy of single-agent therapies with tamoxifen, rIFN-α, or rIFN-β in MPNs.

**Combination Therapy of Tamoxifen, Retinoic Acid, and rIFN-β in MPN?** Early studies in breast cancer showed that all-trans retinoic acid (RA), similar to tamoxifen, was able to upregulate IFN-inducible genes [196]. Similar to combination therapy of rIFN-β and tamoxifen, the combination of rIFN-β and RA has also been shown to exert antiproliferative effects in vitro and in vivo, while also enhancing ISGF-3 activation [196]. Notably, triple therapy with tamoxifen, RA, and rIFN-β has been reported to exert a potentially even stronger antiproliferative effect in breast cancer [197,198,199,200,201,202] and should be pursued in patients with MPNs as well.

## 6. Combination Therapy of a DNA Hypomethylator, BCL-1 Inhibitor, and rIFN-α or rIFN-β?

We have recently proposed a combination therapy of a DNA hypomethylator + ruxolitinib and rIFN-α2 for the treatment of MPN patients in the accelerated phase of MPNs [46]. The rationales for this combined approach are several. First, monotherapy with the DNA hypomethylator azacitidine (Aza) is efficacious in these patients [203], and combination therapy with ruxolitinib may further enhance the efficacy obtained by monotherapy alone [204]. Second, such a combination therapy both directly targets the malignant clone (rIFN-α or rIFN-β + DNA methylator) and dampens the inflammation (ruxolitinib) that fuels the malignant clone. Third, Aza enhances the expression of retroviral proteins, which activate immune signaling through the viral defense pathway, thereby eliciting a type I IFN response and apoptosis [205]. Fourth, the type I IFN response is associated with upregulation and overexpression of hypermethylated endogenous retrovirus (ERV) genes, with ensuing activation of the IFN response [206]. Fifth, by stimulating the expression of retrovirus genes (i.e., virus mimicry), Aza may render MPN cells more immunogenic and, thus, more susceptible to attack by immune cells. Sixth, by enhancing immune cell function, rIFN-α or rIFN-β may further accelerate the killing of MPN cells. Seventh, a recent study has shown that the BCL-1 inhibitor venetoclax directly activates T cells to increase their cytotoxicity against acute myeloid leukemia (AML) in vitro and in vivo [207]. Venetoclax enhanced effector T-cell function by increasing the generation of reactive oxygen species (ROS) [207]. In addition, Aza induced a viral mimicry response in AML cells by activating the STING/cGAS pathway, thereby rendering the AML cells more susceptible to T-cell-mediated cytotoxicity. Similar findings were seen in patients treated with venetoclax, as this treatment increased ROS generation while also activating T cells [207]. Studies on BCL-1 inhibitor treatment of myelofibrosis patients are ongoing. The efficacy of BCL-1 inhibitors in MPNs can likely be attributed to similar mechanisms of action, which should be explored in future studies. Taking into account that both venetoclax and Aza activated T cells, and Aza activated the STING (Stimulator of interferon genes) pathway, it is relevant to consider whether a combination therapy of venetoclax, Aza, and a type I IFN (i.e., rIFN-α or rIFN-β) might further enhance the killing of MPN cells.

## 7. Future Research Directions

Based on 30 years of experience with rIFN-α2 in the treatment of MPNs, showing the safety and efficacy and the recent marketing of the first rIFN-α2b formulation (Besremi) for use in the treatment of newly diagnosed PV patients, we can conclude that stem-cell-targeted therapy with rIFN-α2 will be the cornerstone in the future treatment of MPN patients. Unfortunately, a large number of patients do not tolerate rIFN-α2 or are refractory to treatment. The novel rIFN-α2b Besremi seems to be less toxic and perhaps also more effective than treatment with Pegasys, which is the only alternative today. Therefore, we are in an urgent need of stem-cell-targeting drugs other than Besremi and Pegasys, whether as monotherapies or in combination with agents that target the concurrent chronic inflammatory state, which is considered to be of major importance as the driving force for clonal expansion and evolution in the biological MPN continuum from early cancer stages (i.e., ET and PV) to the advanced myelofibrosis stage. Accordingly, studies on the safety and efficacy of pegylated IFN-β (e.g., Plegridy) are urgently needed, the optimal design being a randomized pilot study between pegylated rIFN-α2 (e.g., Pegasys or Besremi) and rIFN-β, with comparisons of safety, efficacy, and toxicity profiles and concurrent molecular and immune cell studies (i.e., frequencies, distribution, and functionality) before and during treatment. Studies of the safety and efficacy of rIFN-β in patients who are refractory or intolerant to rIFN-α2 might be highly important to determine whether rIFN-β might “rescue“ such patients. Studies of rIFN-β in the CHIP-*JAK2V617F* stage before the overt development of MPNs might also be highly relevant to assess whether rIFN-α2 or rIFN-β might reduce or potentially eradicate the malignant clone in the earliest stages of MPN development [54]. If studies of monotherapy with rIFN-β show similar or even superior safety and efficacy as compared to Besremi or Pegasys, the path is open for studies of the safety and efficacy of the combination therapies mentioned above, and possibly others as well (e.g., hydroxyurea, statins, and colchicine) [54]. Studies on the safety and efficacy of anti-CALR monoclonal antibody therapy are in the pipeline; as part of this research program, it might be tempting to conjugate with rIFN-α2 or rIFN-β. This strategy has been considered for years but has only recently been accomplished in the treatment of multiple myeloma [208,209]. Lastly, in the COVID-19 era, it is important to underscore that several studies have shown rIFN-β to have a favorable impact on the clinical course of COVID-19. The rationales and evidence for using rIFN-β as monotherapy or in combination with ruxolitinib have most recently been thoroughly described [114], and are summarized in Table 2.

## 8. Conclusions and Perspectives

Despite a 30-year history as potent immunomodulatory anticancer agents, the journey of type I IFNs (i.e., IFN-α and IFN-β) has not yet been completed with their successful implementation as safe and efficacious agents to be used routinely in the fight against cancer. Fortunately, at last, a novel pegylated IFN (ropeginterferon-α2b (Besremi) has been launched for marketing to treat patients with PV. Herein, we argue for the rationales and perspectives for initiating clinical studies on the safety and efficacy of rIFN-β -a forgotten drug in the treatment of cancer, but hopefully soon to be revived for the treatment of patients with MPNs, in whom repeated ischemic strokes contribute significantly to morbidity and mortality. From this perspective, repurposing rIFN-β in the treatment of MPNs is expected to open a new horizon for MPN patients, taking into account that rIFN-β may not only be highly efficacious in controlling elevated blood cell counts, but may also play a neuroprotective role—not only against the development of Alzheimer’s disease [129], but also in ischemic stroke prevention [210].

## Figures and Tables

**Table 1:** Some key questions with regard to the impact of rIFN-α2 and rIFN-β on clinical, biochemical, and immunological markers in MPNs.

Impact Upon	rIFN-α2	rIFN-β	Comments/Questions
**Disease-Initiating/** **Propagating Mechanisms**			
**Type I Interferon** **Deficiency**	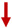	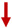	Does treatment with type I IFNs restore the IFN deficiency state in elderly MPN-patients, in whom age-related type I IFN deficiency is prevalent?
**Hyperinflammation**	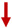	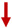	Does treatment with type I IFNs decrease the chronic inflammatory state in MPNs, thereby decreasing the inflammatory drive on the malignant clone? Does the anti-inflammatory potential of rIFN-α2 or rIFN-β protect against progressive COVID-19 infection due to their impact on the hyperinflammatory state and the inflammation-mediated in vivo activation of leukocytes, platelets, and endothelial cells?
**Granulocytosis** **Monocytosis** **Thrombocytosis**	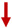	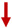	Treatment with rIFN-α2 decreases granulocytosis, monocytosis, and thrombocytosis in MPNs. rIFN-β has been shown to possess antiproliferative capabilities. Thus, the toxicity profile of rIFN-β includes granulocytopenia, monocytopenia, and thrombocytopenia, as shown in several multiple sclerosis studies. Therefore, rIFN-β can likely reduce granulocytosis, monocytosis, and thrombocytosis in MPNs. Does type I rIFN therapy protect against progressive COVID-19 infection due to its impact on granulocyte counts and inflammation-mediated in vivo activation of leukocytes, platelets, and endothelial cells?
**Thrombosis**	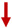	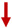	Does treatment with rIFN-α2 or rIFN-β reduce the risk of thrombosis?
**NETosis**	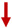	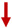	Does treatment with rIFN-α2 or rIFN-β inhibit NETosis formation?

**Table 2:** Rationales for treatment with rIFN-α2 and rIFN-β in patients with MPNs and COVID-19.

Biomarker	Impact	Comments
Viral Replication	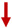	rIFN-α2 and rIFN-β are highly potent antiviral agents [108,109,110,111,112,113,114,115,130,131,132,133,134,135,136]
Type 1 IFN Deficiency	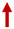	rIFNs restore the IFN deficiency state, thereby impairing viral replication and viral shedding [108,109,110,111,112,113,114,115]
Immune Response	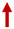	rIFNs strongly boost virtually all immune cells (e.g., dendritic cells, B cells, T cells, NK cells), thereby impairing viral replication and viral shedding [108,109,110,111,112,113,114,115,118,119,120,121,122,123,124,125,126,130,131,132,133,134,135,136,153]
Hyperinflammation	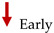 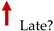	Through the impairment of viral replication, rIFNs alleviate the primary trigger and driver of the cytokine storm; this holds true in the early disease stage. If administered during the cytokine storm, rIFNs may “fuel the fire” and aggravate clinical deterioration, although this issue is controversial [108,109,110,111,112,113,114,115]
Thrombosis Risk	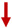 ?	rIFN-α2 normo- or downregulates upregulated thromboinflammatory genes, including PAD4, which mediates NETosis (to be submitted)
**Clinical Improvement**		
COVID-19	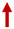	Several studies have reported clinical improvement during treatment with either rIFN-α2 or rIFN-β [112,113,114,115]
Chronic Blood Cancers (ET, PV, and Myelofibrosis) (MPNs)	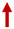	Excellent safety and efficacy profiles: rIFN-α2 normalizes elevated cell counts within weeks to months, which can be explained by several mechanisms, including directly targeting the malignant stem cells (or targeting SARS-CoV-2 in COVID-19) in concert with boosting of immune cells and upregulation of downregulated (inflammation-mediated?) HLA genes [22,31,32,33,34,35,36,37,38,39,40,41,42,43,44,45,46,47,48,49,50,51,52,53,54,68,69,70,71,72,73,74,75,76,77,78,79,80,81,82,83,84,85,86,87,88,89,90,91,92,93,94,95,96,97,98,99,100,101,102]
Hepatitis B and C	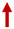	Excellent safety and efficacy profiles: For decades, rIFN-α2 (rIFN-α2a or rIFN-α2b) has been one of the standard treatments for hepatitis B and C [108,109,110]
Multiple Sclerosis	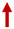	Excellent safety and efficacy profiles: For decades, rIFN-β has been one of the standard treatments for MS [104,105,106,107]

Abbreviations: IFN = interferon; ET = essential thrombocythemia; PV = polycythemia vera.

## Data Availability

Not applicable.
